# The Influence of Insulin Dependent Diabetes Mellitus on Dental Caries and Salivary Flow

**DOI:** 10.1155/2014/790898

**Published:** 2014-10-14

**Authors:** V. K. Gupta, Seema Malhotra, Vasuda Sharma, S. S. Hiremath

**Affiliations:** ^1^Department of Public Health Dentistry, FODS, KGMU, Lucknow 226003, India; ^2^Department of Pedodontics, Saraswati Dental College and Hospital, Lucknow 227105, India; ^3^Department of Public Health Dentistry, RajaRajeswari Dental College & Hospital, Bangalore 560074, India; ^4^Department of Public Health Dentistry, The Oxford Dental College, Bangalore 560068, India

## Abstract

*Objective*. To assess whether or not there was any change in the dental caries and rate of salivary flow of patients with Insulin Dependent Diabetes Mellitus (IDDM) and the contribution of salivary flow to caries risk in IDDM. *Setting*. Department of Endocrinology, MS Ramaiah Hospital, Bangalore, India. *Design*. A comparative cross-sectional descriptive type. *Materials and Methods*. The sample consisted of two groups: 140 diabetic group (mean age 14.8 yr) and 140 nondiabetic group (mean age 13.7 yr). Dental caries by dmf(t) and dmf(s) indices for primary dentition and DMF(T) and DMF(S) indices was used in permanent dentition to assess the dental caries experience. Both stimulated and unstimulated salivary flow rate were assessed after collection of saliva. *Results*. In diabetic group 76% had carious lesion and in nondiabetic group 85.3% had carious lesion. Diabetics have lower mean DMFT, DMFS, dmft, and dmfs compared to the nondiabetic group. Diminished unstimulated and stimulated salivary flow rate in diabetic than nondiabetic group.* Conclusions*. The findings obtained conclude that even though there was reduced salivary flow rate in diabetic group the caries prevalence was low.

## 1. Introduction

Diabetes mellitus is a chronic disease resulting from a relative or absolute deficiency of insulin, which affects the metabolism of carbohydrate, protein, and fat. The most obvious abnormality is a high level of blood glucose especially following a meal.

According to data of World Health Organization DIAMOND Project Group, while the incidence of type 1 diabetes mellitus was low in Asia and South America and high in Europe [1], in India many hospital records and clinic data indicate that young diabetics (diabetes onset before 15 years) constitute 1–5% of the total diabetic subjects enrolled. South India has a lower incidence of 10.5/100,000/year [1].

The current concept in diabetic care with blood glucose monitoring and frequent injections of short-acting insulin allows a less restricted diet [2]. This may affect oral health rapidly and therefore requires attention. However, there are many internal and external factors which might contribute to diabetes mellitus and in turn affect the general health and more so oral health.

Inflammatory disease of the periodontium considered as the sixth complication of diabetes mellitus [3]. The influence of diabetes on the risk of developing increased prevalence of dental caries in patients with type 1 diabetes has been the subject of much discussion in literature. On one hand, some authors [4] have reported fewer caries in type 1 diabetic patients. However, other authors have reported an increased presence of caries in diabetic patients [5].

The oral cavity is constantly exposed to saliva whose important function is to dilute and oral clearance. Diabetes may cause changes in salivary glands, which may contribute to slow flow rate and alter saliva composition.

The aim of this study was to assess whether or not there was any change in the dental caries and rate of salivary flow of patients with IDDM and the contribution of salivary flow to caries risk in Insulin Dependent Diabetes Mellitus subjects.

## 2. Material and Methods

The study group consisted of a sample of 140 subjects, ranging in age from 10 to 15 years who were registered at the Department of Endocrinology, MS Ramaiah Hospital, for treatment of their type 1 diabetes mellitus. In the second group there were 140 young subjects, ranging in age from 10 to 15 years who were not suffering from diabetic or any systemic disease. The subjects selected in the second group were siblings, neighbors, and friends of these patients thus reflecting the same social strata. All diabetic children were on insulin therapy and data on blood glucose level and glycosylated hemoglobin (HbA1c) were collected from department record.

The study design was approved by Ethical Committee of Government Dental College and Research Centre. All subjects and their families were informed about the method and purpose of the study and informed consent was obtained from parents of those who participated in the study.

A pilot study has been conducted to determine sample size, feasibility, intraexaminer variability, and so forth. Pilot survey revealed many initial carious lesions among IDDM patients; hence, WHO criteria for assessment of dental caries were not used.

Criteria used for assessment of dental caries in primary dentition are dmft (Decayed/Missing/Filled Teeth) and dmfs (Decayed/Missing/Filled Surface) indices given by Greubbell [6] and in permanent dentition are DMFT (Decayed/Missing/Filled Teeth) and DMFS (Decayed/Missing/Filled Surface) indices given by Klein et al. [7].

Recording of the intraoral examination was carried out at the Department of Endocrinology by a single calibrated examiner under natural light using a sterile mouth mirror and explorer on ordinary chair.

The subjective experience of dryness of mouth was diagnosed by means of the question: “Do you feel that your mouth is dry frequently?”

Salivary flow rate was determined by asking study participants to swallow any accumulated saliva and to clear the mouth prior to collection of saliva. The participants are requested to relax for 5 minutes and not to eat, drink, chew gum, or brush the teeth 1 hour prior to this procedure. Unstimulated salivary flow (USF) rate was determined by asking the study participants to passively drool into a funnel inserted into a graduated cylinder for 5 minutes. The volume of saliva collected in the graduated cylinder after 5 minutes is divided by 5 to determine the USF. The salivary flow rate was calculated in mL/min. A USF rate less than 0.1 mL per minute is diagnostic of salivary gland hypofunction. Stimulated salivary flow was determined after asking the study participants to chew unflavored paraffin wax for 1 minute [8].

### 2.1. Statistical Analysis

Data were analysed using statistical package for social science (SPSS) version 13. The chi-square test was used to compare proportion and one-way analysis of variance was used to compare the means. Difference between the study groups was assessed by Student's* t*-test.

## 3. Results

The diabetic group consisted of 140 subjects (52.7% were males and 47.3% were females), mean age 14.8 yr, and in nondiabetic group there were 140 healthy subjects (44.7% were males and 55.3% were females), mean age 13.7 yr.

**Figure 1:**
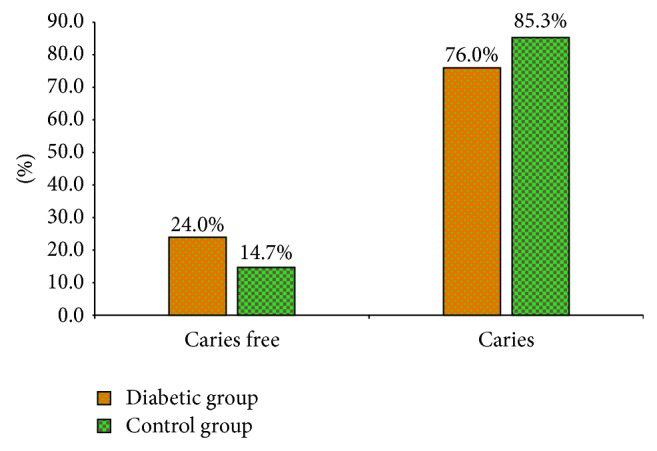
Prevalence of dental caries in both groups.

The prevalence of dental caries in diabetic group was statistically (*P* = 0.041) lesser than nondiabetic group as shown in Figure 1.

The mean number of Decayed, Missing, and Filled Teeth (DMFT) was statistically (*P* = 0.004) lesser in diabetic control groups (2.09) compared to nondiabetic groups (2.25). The mean number of decayed teeth (DT) was statistically (*P* = 0.005) lesser in diabetic group (1.91) compared to nondiabetic groups (2.07). The mean number of missing teeth (MT) was statistically (*P* = 0.0) lesser in diabetic group (0.047) compared to nondiabetic groups (0.04) as shown in Table 1.

The mean number of Decayed, Missing, and Filled Surfaces (DMFS) was less in diabetic group (2.25) compared to nondiabetic group (2.74). The mean number of decayed surfaces (DS) was less in diabetic group (2.05) than in nondiabetic group (2.43). The mean number of missing surfaces (MS) was slightly higher in diabetic group (0.19) than in nondiabetic group (0.16). The mean number of filled surfaces (FS) was significantly (*P* = 0.002) lower in diabetic group (0.02) than in nondiabetic group (0.15) as shown in Table 1.

The mean number of Decayed, Missing, and Filled Teeth (dmft) was less in diabetic group (0.59) compared to nondiabetic group (0.77). The mean number of decayed teeth (dt) was less in diabetic group (0.47) compared to nondiabetic group (0.75). The mean number of missing teeth (mt) was less in diabetic group (0.007) compared to nondiabetic group (0.0). Statistically significant difference (*P* = 0.001) was found in mean no. of filled teeth (ft) in diabetic group (0.12) than in nondiabetic group (0.02) as shown in Table 2.

The mean number of Decayed, Missing, and Filled Surfaces (dmfs) was statistically (*P* = 0.006) lower in diabetics (0.6) than in nondiabetic group (1.15). The mean number of decayed surfaces (ds) was statistically (*P* = 0.007) lower in diabetic group (0.57) than in nondiabetic group (1.14). The mean number of filled surfaces (fs) was lower in diabetic group (0.027) than in nondiabetic group (0.013). Statistically significant difference was found in mean dmfs and ds of diabetic and nondiabetic group as shown in Table 2.

In the category of less than 0.1 mL/min unstimulated salivary flow, the mean dmf(t), dmf(s), DMF(T), and DMF(S) were less in diabetic group (0.48, 0.5, 2.28, and 2.41, resp.) than in nondiabetic group (0.62, 1.0, 4.0, and 4.75, resp.) and statistically significant difference (<0.05) was found between mean DMF(T) of diabetic and nondiabetic group as shown in Table 3.

In the category of 0.1–0.5 mL/min unstimulated salivary flow, the mean dmf(t), dmf(s), DMF(T), and DMF(S) were less in diabetic group (0.66, 0.66, 1.96, and 2.16, resp.) than in nondiabetic group (0.78, 1.16, 2.14, and 2.62, resp.) and statistically significant difference was found in mean dmfs and DMFS of diabetic group and nondiabetic group as shown in Table 3.

In the category of less than 0.1 mL/min stimulated salivary flow, the mean dmf(s), DMF(T), and DMF(S) were less in diabetic group (0.80, 2.77, and 3.08, resp.) than in nondiabetic group (1.0, 3.66, and 5.0, resp.), but the mean dmf(t) was more in diabetic group (1.75) than in nondiabetic group (0.58) as shown in Table 4.

In the category of 0.1–0.5 mL/min stimulated salivary flow, the mean dmf(t), dmf(s), DMF(T), and DMF(S) were less in diabetic group (0.32, 0.24, 2.16, and 2.22, resp.) than in nondiabetic control group (1.6, 2.33, 3.26, and 4.06, resp.) and statistically significant difference was found in mean dmf(t), dmf(s), DMF(T), and DMF(S) of diabetic group and nondiabetic group as shown in Table 4.

In the category of 0.6–1.0 mL/min stimulated salivary flow, the mean dmf(t), dmf(s), DMF(T), and DMF(S) scores were less in diabetic group (0.73, 0.77, 0.038, and 0.038, resp.) than in nondiabetic control (0.66, 1.0, 2.24, and 2.68, resp.) and statistically significant difference was found in mean DMF(T) and DMF(S) of diabetic group and nondiabetic group as shown in Table 4.

Statistically significant difference was found regarding dryness of mouth (xerostomia) between diabetics group (30%) of patients and control group (6.67%) subjects.

## 4. Discussion

IDDM is one of the most common chronic diseases of childhood. Its implication on periodontium is very well documented. Dental caries is much less investigated and the results have been notably inconsistent compared to periodontal disease in subjects with diabetes.

In the present study, the prevalence of dental caries was found to be less in diabetic group than in the nondiabetic group, which is similar to the literature finding [9–11]. Few studies [12, 13] have shown comparatively greater prevalence of caries among patients with diabetics. Many literatures describe no difference in the prevalence of caries between diabetics and nondiabetics [14–18].

The mean value of DMFT among diabetic group (2.09) was lower than the nondiabetic group (2.25). Literatures [9, 12, 18] have shown that the diabetic group showed higher mean (17.7, 20.1, and 4.3, resp.) values than nondiabetic group (14.9, 17.4 and 3.3, resp.).

The mean value of DMFS among diabetic patients (2.25) was lower than the nondiabetic group (2.74). Literatures [18–20] have shown that diabetic group had higher mean (5.2, 5.8, and 33.7, resp.) values than nondiabetic group (5.0, 4.0, and 26.2, resp.).

Increased risk of dental caries would be related to certain factors such as poor oral hygiene or a lack of blood glucose control. This latter situation could be due to an incorrect diet or deficient control of the administered insulin dose with respect to physical exercise of the timing of meals [21, 22].

It has been suggested that hyperglycemia in diabetes is associated with decreased salivary secretion and high salivary glucose levels, due to disturbed glucose metabolism [23, 24]. Moreover, dry mouth was a major complaint of many IDDM patients. Literature [25] in support shows that both unstimulated and stimulated salivary flow rates are reduced in diabetic patients, whereas literature [26] reported that only unstimulated salivary flow is reduced and in contrast literatures [27–29] did not find any significant differences in salivary flow rates between diabetic and nondiabetic individuals.

Our finding showed that in subject with low unstimulated salivary flow rate the mean DMFT was found to be significantly lower in diabetic group than in nondiabetic group. This could be because in diabetic subject the good metabolic control prevents the most dangerous salivary changes such as high glucose content and lower pH and a good diabetic diet, rich in fiber and low in simple carbohydrates, can slow down the production of plaque and the proliferation of acidogenic bacterial microflora [30, 31].

*Study Limitation*. The information collected from the camps of IDDM children has been regularly organized by the department. Investigator did not collect the information regarding the well-controlled and poorly controlled diabetes which may affect DMFT/dmft/DMFS/dmft values. The author may suggest further studies.

## 5. Conclusion

Dental caries is an infectious disorder involving multiple factors that coincide at a given point and at a given time. The clinical results of the present study indicate decreased vulnerability to dental caries in patients with IDDM compared with nondiabetic group.

## Figures and Tables

**Table 1:** Distribution of study participants with mean D(T), M(T), F(T), DMF(T), D(S), M(S), F(S), and DMF(S) according to study group.

Variable	Diabetic group (*n* = 140) mean ± SD	Nondiabetic Group (*n* = 140) mean ± SD	*P* value
DT	1.91 ± 1.94	2.07 ± 1.6	0.005
MT	0.047 ± 0.21	0.04 ± 0.20	0.00
FT	0.13 ± 0.38	0.14 ± 0.45	0.84
DMFT	2.09 ± 2.00	2.25 ± 1.64	0.004
DS	2.05 ± 2.18	2.43 ± 1.99	0.128
MS	0.19 ± 0.85	0.16 ± 0.79	0.759
FS	0.02 ± 0.14	0.15 ± 0.47	0.002
DMFS	2.25 ± 2.31	2.74 ± 2.11	0.064

**Table 2:** Distribution of study participants with mean d(t), m(t), f(t), dmf(t), d(s), m(s), f(s), and dmf(s) according to study group.

Variable	Diabetic group (*n* = 140) mean ± SD	Nondiabetic group (*n* = 140) mean ± SD	*P* value
dt	0.47 ± 1.32	0.75 ± 1.35	0.08
mt	0.007 ± 0.082	0	0.313
ft	0.12 ± 0.37	0.02 ± 0.14	0.001
dmft	0.59 ± 1.36	0.77 ± 1.37	0.27
ds	0.57 ± 1.64	1.14 ± 1.89	0.006
ms	0.008 ± 0.084	0	0.317
fs	0.027 ± 0.16	0.013 ± 0.12	0.408
dmfs	0.6 ± 1.63	1.15 ± 1.91	0.007

**Table 3:** Distribution of study participants with mean dmf(t), dmf(s), DMF(T), and DMF(S) according to unstimulated salivary flow.

Unstimulated salivary flow		dmft	dmfs	DMFT	DMFS
Less than 0.1 mL/min	Diabetic group (*n* = 56)	0.48	0.5	2.28	2.41
	Nondiabetic group (8)	0.62	1.0	4.0	4.75
	*P* value	0.737	0.413	0.012	0.003

0.1–0.5 mL/min	Diabetic group (84)	0.66	0.66	1.96	2.16
	Nondiabetic group (132)	0.78	1.16	2.14	2.62
	*P* value	0.520	0.04	0.459	0.119

**Table 4:** Distribution of study participants with mean dmf(t), dmf(s), DMF(T), and DMF(S) according to stimulated salivary flow.

Stimulated salivary flow		dmft	dmfs	DMFT	DMFS
<0.1 mL/min	Diabetic group (71)	1.75	0.80	2.77	3.08
	Control group (3)	0.58	1.0	3.66	5.0
	*P* value	0.938	0.876	0.493	0.224

0.1–0.5 mL/min	Diabetic group (53)	0.32	0.24	2.16	2.22
	Control group (30)	1.6	2.33	3.26	4.06
	*P* value	0.00	0.00	0.005	0.00

0.6–1 mL/min	Diabetic group (26)	0.73	0.77	0.038	0.038
	Control group (98)	0.66	1.0	2.24	2.68
	*P* value	0.767	0.469	0.00	0.00

>1 mL/min	Diabetic group (0)	0	0	0	0
	Control group (19)	0.66	0.51	0.35	1.0
